# Diversification of immunoglobulin genes by gene conversion in the domestic chicken (*Gallus gallus* domesticus)

**DOI:** 10.1093/discim/kyad002

**Published:** 2023-01-19

**Authors:** Jessica Mallaby, William Mwangi, Joseph Ng, Alexander Stewart, Daniel Dorey-Robinson, David Kipling, Uri Hershberg, Franca Fraternali, Venugopal Nair, Deborah Dunn-Walters

**Affiliations:** Department of Bioscience and Medicine, University of Surrey, Guildford, UK; Pirbright Institute, Woking, UK; Pirbright Institute, Woking, UK; Randall Centre for Cell and Molecular Biophysics, King’s College London, London, UK; Department of Bioscience and Medicine, University of Surrey, Guildford, UK; Pirbright Institute, Woking, UK; Department of Bioscience and Medicine, University of Surrey, Guildford, UK; Department of Human Biology, University of Haifa, Haifa, Israel; Randall Centre for Cell and Molecular Biophysics, King’s College London, London, UK; Pirbright Institute, Woking, UK; Department of Bioscience and Medicine, University of Surrey, Guildford, UK

**Keywords:** B Cell, Gene Conversion, Avian, Immunology

## Abstract

Sustainable modern poultry production depends on effective protection against infectious diseases and a diverse range of antibodies is key for an effective immune response. In the domestic chicken, somatic gene conversion is the dominant process in which the antibody immunoglobulin genes are diversified. Affinity maturation by somatic hypermutation (SHM) also occurs, but the relative contribution of gene conversion versus somatic hypermutation to immunoglobulin (Ig) gene diversity is poorly understood. In this study, we use high throughput long-read sequencing to study immunoglobulin diversity in multiple immune-associated tissues in Rhode Island Red chickens. To better understand the impact of genetic diversification in the chicken, a novel gene conversion identification software was developed (BrepConvert). In this study, BrepConvert enabled the identification of over 1 million gene conversion events. Mapping the occurrence of putative somatic gene conversion (SGC) events throughout the variable gene region revealed repetitive and highly restricted patterns of genetic insertions in both the antibody heavy and light chains. These patterns coincided with the locations of genetic variability in available pseudogenes and align with antigen binding sites, predominately the complementary determining regions (CDRs). We found biased usage of pseudogenes during gene conversion, as well as immunoglobulin heavy chain diversity gene (IGHD) preferences during V(D)J gene rearrangement, suggesting that antibody diversification in chickens is more focused than the genetic potential for diversity would suggest.

## Graphical Abstract

**Graphical Abstract:**
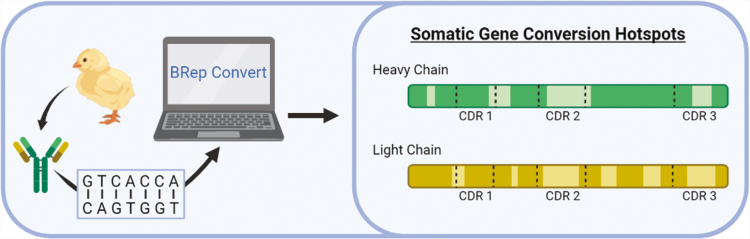

## Introduction

The domestic chicken (*Gallus gallus* domesticus) is an agriculturally important species, as it is the most efficient producer of animal protein [1]. In the UK the poultry industry contributes to 14.5% of the total UK agricultural industries net worth, 11.2% from poultry meat production and 3.3% from egg production [2]. Despite this, there is much still to learn about chicken immunological mechanisms.

In chickens, lymphocytes start as haematopoietic stem cells originating from the yolk sac, these cells differentiate into lymphocyte progenitor cells and migrate towards the embryonic spleen where they undergo V(D)J gene rearrangement. Unlike mammalian species V(D)J gene rearrangement in the chicken occurs in the heavy and light chains simultaneously [3, 4]. Following the successful rearrangement of variable (V), diversity (D), and joining (J) gene segments, these cells become B cell progenitors and migrate into the bursa of Fabricius. Once inside the bursa, these cells colonize the bursal follicles, and undergo proliferation and further diversification by SGC and SHM [5].

Avian species have a small selection of immunoglobulin genes during V(D)J gene rearrangement. Domestic chickens only possess a single functional heavy chain variable (IGHV) gene and a heavy chain joining (IGHJ) gene. When these genes are brought together with a heavy chain diversity (IGHD) gene to form a functional heavy chain immunoglobulin gene (IGH), the level of diversity created is very limited, particularly in the light chain (IGL) where the immunoglobulin gene is comprised of only variable and joining genes. To counteract this somatic gene conversion (SGC) and somatic hypermutation (SHM) are employed to further diversify the variable gene [6, 7]. SGC is activated in the functional immunoglobulin gene via the deamination of cytidine nucleotides by the activation-induced cytidine deaminase (AID) enzyme, and the generation of double-strand breaks. SGC repairs these breaks in the genetic sequence using variable region pseudogenes. Pseudogenes are located upstream of the functional variable, diversity and joining genes and are genetically similar to functional IGHV genes, however, they do not possess recombination signal sequences, and as result they are unable to be incorporated during V(D)J gene rearrangement. The domestic chicken has a much larger library of variable region pseudogenes in comparison to functional variable genes, and SGC utilizes these pseudogenes as genetic templates to repair double-strand breaks in the functional immunoglobulin gene [7, 8]. Evidence has been found to show that other agricultural species such as cattle, sheep, and horses also employ the use of SGC. But due to a lack of diverse variables and joining gene segment libraries, chickens are especially reliant on the secondary gene diversification processes [9, 6]. Functional immunoglobulins also undergo diversification by SHM, in which the AID enzyme also plays a role. But during SHM cytidine deamination events are simply replaced by another nucleotide, resulting in a single point mutation.

The variable gene region can be subdivided into five functional regions based on tertiary structures three framework regions (FWR) and two complementary determining regions (CDR). A third CDR spans across all three V(D)J regions in the heavy chain and across VJ regions in the light chain and is therefore highly diverse. During protein folding loops are formed in the immunoglobulin gene, these loops are expressed on the outside of the protein and form the antigen binding site, and are comprised of the three CDRs. Contrastingly, the three FWRs are internalized during protein folding and play a key role in maintaining the three-dimensional structure [10–12]. Chickens produce three classes of antibodies, Immunoglobulin A (IgA), Immunoglobulin M (IgM), and Immunoglobulin Y (IgY). Similarities can be found between avian IgA and IgM and their mammalian counterparts, whereas avian IgY antibodies share similarities with both mammalian IgG and IgE [13].

In this study, we amplified transcripts coding for the B cell repertoire of six Rhode Island Red chickens and used high throughput long-read sequencing to generate an immunoglobulin gene library. This was used to conduct an depth analysis of somatic gene conversion events and identify how they manifest within the variable gene. Analysis of gene conversion at this scale has previously been restricted due to the lack of available analysis tools. For that reason, we designed an automated computational pipeline (BrepConvert) that could identify and record multiple gene conversion events across thousands of sequences. To aid future analysis of gene conversion this R package has been made publicly available. We also developed a novel way of visualizing this information by mapping the location of gene conversion events across the variable gene region to identify patterns of insertion.

## Materials and methods

### Sampling

The bursa of Fabricius, cecal tonsil, and spleen were collected from six 3-week-old Rhode Island Red chickens. All tissue samples were processed individually immediately after collection. To extract the lymphocyte cells, bursal tissue, and the cecal tonsil required an initial incubation step in 1 ml of 8 mg/ml of collagenase D diluted in HBSS for 1 h and 30 min with agitation. All tissues were then crushed though a 40 µm cell strainer before being overlayed onto histopaque (Merck – 10771) at a 2:1 ratio respectively. After extraction lymphocytes were counted using a haemocytometer, resulting the extraction of between 15 and 60 × 10^6^ cells from bursal tissue, 10–30 × 10^6^ from spleen tissue and 0.5–3 × 10^6^ from cecal tonsil tissue. Qiagen RNeasy Mini Kit (Qiagen – 74104) was used to extract RNA from the total lymphocyte population collected from each tissue sample, following the manufacturer’s instructions, all samples were then quality checked using a Nanodrop.

### Reverse transcription and PCR amplification

Samples were converted from RNA, 170 ng minimum, to cDNA using SMARTScribeTM reverse transcriptase (Clontech) following the manufacturer’s protocol and the SmartNNN TSO primer (Supplementary Table 1a). Samples were then treated with 0.5 µl of Uracil DNA Glycosylase (UDG) (NEB – M0280S) and incubated for 1 h at 37ºC and then for 10 min at 95ºC. The samples were treated with UDG to prevent interference with unique molecular identifiers (UMIs). This was followed by two semi-nested and semi-step out PCR reactions, using Q5 High Fidelity Polymerase (NEB – M0492S) following the manufacturers’ protocol with an annealing temperature of 65ºC for 20 s and an extension temperature of 72ºC for 50 s. These PCR steps were semi-nested due to the use of nested PCR primers at the 3’, and semi-step out due to the use of primers that insert additional primer landing sites at the 5’. Each of the antibody classes (IgA, IgM, IgY and Lambda light chain (IgL)) were amplified in separate 20 µl reactions. To reduce biased amplification of individual mRNA sequences four separate reactions were amplified for each antibody class, 1 µl of each replicate was then carried over into a further four reactions, resulting in a total 16 final reactions per antibody class (64 final reactions per sample). The first PCR reaction utilized the Smart20 forward primer and a class specific reverse primer (Supplementary Table 1b), for a total of 21 cycles. The second PCR reaction used a step out patient identifier (PID) forward primer and the respective antibody class specific nested reverse primer and the respective antibody specific reverse primer (Supplementary Table 1c) for a total of 15 cycles. The step out PID primer used in the final PCR, inserted a known sequence at the 5’ end of the amplified immunoglobulin. Each tissue was given a unique step out PID primer, so that during data analysis, the origin of the sample could be identified. Following this all samples were analysed using a bioanalyzer (Agilent 7500), all 16 reactions for each antibody class were then pooled in equal concentrations into a single aliquot. These were then cleaned using a Wizard PCR Clean up kit (Promega – A9281) following the manufacturers protocol and eluting into 30 µl. Using a PippinPrep with marker K (SAGE Bioscience), DNA was extracted and purified from each sample, in this process DNA bands were extracted based on expected length described on the international immunogenetics information system (IMGT) [14]. The DNA was then quantified using the Qubit DNA quantification kit following the manufacturers protocol, before all four antibody classes from each tissue sample were pooled. The final purification step was completed using SPRI select beads (Beckman Coulter – B23318) at a concentration of 0.8× the samples volume, with a final elution of 30 µl. These samples were sent to the Centre for Genomic Research at Liverpool University for PacBio NGS sequencing. For full details of methods see [15].

### Preparation of PacBio sequencing data

The subreads generated by PacBio NGS were processed into circular consensus sequences (CCS), with a minimum requirement of 10 subreads, resulting in a total of 341,115 consensus sequences. UMIs were identified in 86% of all sequences generated through PacBio, on average 98% of the UMIs were identified to have less than or equal to 10 sequences per UMI (Supplementary Fig. 1). In cases where multiple sequences were identified to have the same UMI, the sequences were collapsed to generate a CCS. Bird ID numbers and tissues of origin were assigned by identifying the PID sequences using agrep (TRE agrep V0.8.0) and allowing for up to 3 nucleotide mismatches. Sequences that were found to not contain a PID sequence were removed from analysis. The antibody class was identified using immunoglobulin constant region motifs listed on IMGT. Only 143,034 sequences remained after the removal of sequences that did not contain valid UMIs or PID sequences and the collapsing of multiplicated UMIs. These sequences were then characterized and cleaned using IMGT High V Quest [14], removing only 1% of sequences due to incomplete variable gene sequences. Then finally the data was processed using BRepertoire online software (v1.2.0) [16], to identify a wide variety of sequence characteristics and to calculate the physiochemical properties of each sequence.

### Pseudogene diversity analysis

Diversity and richness metrics designed by [17] to calculate genetic diversity at both the amino acid and nucleotide level, was used to analyse the diversity of each nucleotide position of heavy and light chain pseudogenes. This analysis generated values ranging between 1 and 4 for each nucleotide position illustrating the variety of nucleotide bases.

### BrepConvert — identification of gene conversion events

**Figure 1:**
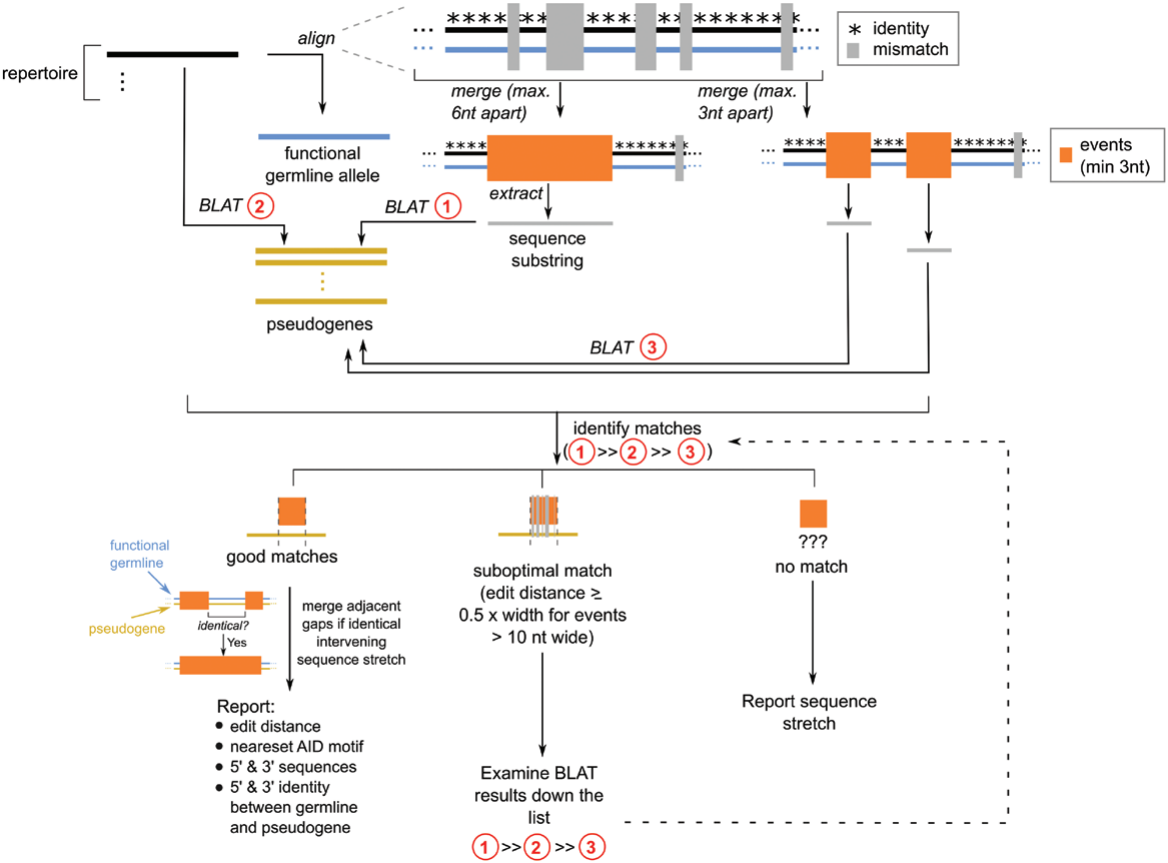
Schematic to illustrate the bioinformatics pipeline used to annotate gene conversion events. Sequences sampled in the repertoire (top left, black thick lines) are separately aligned to the functional germline allele (blue thick lines) to identify regions of mismatches (grey rectangles). Mismatched sequence stretches which are close to one another are merged as ‘events’ which are taken as candidate gene conversion events. Here, two definitions (3 nucleotides (nt) or 6 nt) are used to consider definitions of conversion events of varying stringency. These candidate conversion events are then aligned to the pseudogene DNA sequences (yellow thick lines) using BLAT; analogous alignments of the repertoire sequences to pseudogenes are performed to ensure pseudogenes that match short events are covered. The alignment results are parsed to identify pseudogenes matched to every nominated gene conversion event; events with suboptimal matches (bottom, middle branch) are reconsidered for shorter definitions of the events in order to seek pseudogenes with adequate similarity to the observed converted sequence. The conversion events are further pruned to merge adjacent events separated by identical sequence stretches, and further annotated for the local DNA sequence contexts. Sequence stretches without matching functional and pseudogene alleles are reported separately. For colour figure refer to online version.

BrepConvert was developed as a pipeline for the computational annotation of gene conversion events for each sampled immunoglobulin sequence in the repertoire. This reported: (a) the position (start and end points) of gene conversion events; (b) the pseudogene involved and; (c) the implicated stretch of DNA and protein sequence annotated, from both the converted sequence and the functional allele (Fig. 1). Sequencing data from each of the six individual birds was analysed separately using BrepConvert, the resulting data showed that on average 96% of immunoglobulin sequences were found to include gene conversion events, ranging between 95% and 99% across the six individuals. DNA sequences of functional and pseudogene alleles for the chicken IgH and IgL loci were obtained from the IMGT database. Intuitively, gene conversion events could be identified by aligning the observed sequence with the functional allele, reporting mismatched stretches as conversion events; these stretches could be subject to a subsequent alignment against pseudogenes to identify the ‘donors’ of the conversion, here taken as pseudogenes with highest similarity to the observation. In some cases, a 100% sequence match was not obtained due to occurrence of suspected SHM events and the potential for polymorphic differences between breeds and individuals. We first noted that, due to local similarities between DNA sequences of the functional allele and pseudogenes, a gene conversion event involving a long sequence stretch may not show up as a continuous stretch of positions with mismatches when aligning the observed sequence with the functional allele. Instead, shorter mismatched sequence stretches are interspersed with regions of complete sequence identity. Therefore, we adopted the following heuristic approach to identify gene conversion events for a given observed DNA sequence in the repertoire. We first performed a pairwise alignment (using the Biostrings::pairwise Alignment function in R) between the observed sequence with the functional V-gene allele. This identifies short stretches of mismatches (hereafter termed ‘events’; minimum 3 nucleotides long) along the alignment. No maximum length for gene conversion events was included, due to the potential for events to ranging up 300 nucleotide bases long [18]. Next, we merged ‘events’ together if they were within x nucleotides apart, to form candidate definitions of gene conversion events. Two values of x were arbitrarily chosen: *x* = 6 and *x* = 3; the former would merge separate ‘events’ which are further apart, and therefore would likely propose longer conversion events at the expense of grouping unrelated conversions. This is addressed with the stricter definition (*x* = 3) so that these ‘events’ could be considered separately. Note in either case the gene conversion events were defined as regions of sequence mismatches (or regions flanked by sequence mismatches) when compared against the functional allele; the actual length of sequence stretches implicated in gene conversion could be longer, as they could start and end at regions identical to the functional allele. We addressed this by providing alternative, longer, definitions by counting the length of sequence stretches surrounding the ‘events’ which were identical between the functional and pseudogene alleles (see below).

### Annotating donor pseudogenes

The candidate gene conversion events were then subjected to alignment against the set of pseudogenes as annotated by IMGT. Alignment was performed using the BLAT software tool (v.36x4) with the following additional settings: -out = blast8 -tileSize = 6 -stepSize = 1 -minScore = 20 [19]. A total of three sets of alignments were considered: (a) alignment of events defined by merging ‘gaps’ 6 nucleotides apart; (b) alignment of the full-length observed V-gene sequence against the pseudogene database and; (c) alignment of events defined by merging ‘gaps’ 3 nucleotides apart. We observed that using only the partial sequence stretches in the ‘gaps’ is adequate in annotating the donor pseudogene for most cases, but occasionally using solely the ‘gaps’ failed, conceivably due to limited sequence length being insufficient to trigger an alignment; in these cases, alignment using the full-length observed V-gene sequence could compensate and provide candidate donor pseudogenes. For each gene conversion event, the aligned pseudogenes were ordered by their identity to the ‘gap’ sequence; the best-match pseudogene was nominated as the donor pseudogene. The Levenshtein distance (i.e. number of edits required to convert from the pseudogene to the observed sequence) specific to the gene conversion event was reported 20.

### Pruning and further annotation

We sought further refinement and annotation of the identified gene conversion events. ‘Gaps’ with suboptimal pseudogene alignments (defined here as having Levenshtein distance > 0.5w, where w is the width of the ‘gap’ aligned; suboptimal alignments were considered only for w > 10) were searched through alignments using other definitions of ‘gaps’ as detailed in the previous section ‘Annotating donor pseudogenes’ to seek better pseudogene matches within the range stated above. Gene conversion events without optimally aligned pseudogenes nominated after searching through all performed alignments were noted separately. We reasoned that the sequence stretches next to the nominated gene conversion event and identical between the functional allele and the nominated pseudogene, could be part of the same conversion but missed due to the limitation of focusing on identifying mismatches. We, therefore, pruned the nominated gene conversion events to merge adjacent conversion events separated by a sequence stretch that is identical between the functional and pseudogene alleles. Moreover, the lengths of identical sequence stretch at either end were reported; this constitutes the longest possible definition of gene conversion events, as opposed to the short definition based on mismatches.

We further provided the following annotations to each event: first, we parsed the local DNA sequence context of the gene conversion event by extracting 10 nucleotides in the functional allele 5’ and 3’ to the event. Second, the 5’ distance to the nearest trinucleotide AID target motif (any one of the following: AGC, AGT, GGC, GGT, TGC, AAC, TAC) is reported [21–23]. The identified gene conversion events and associated annotations were exported as a spreadsheet for further data analysis.

### Calculation of PCR crossover and template jumping

In order to estimate the false discovery rate of gene conversion events we performed BrepConvert on human sequences, which are widely considered not to gene convert at a readily detectable rate [24]. Previous research has identified the presence of gene conversion-like events in human immunoglobulin sequences; however, it has been concluded that these events are rare and occur infrequently in humans [25]. Specifically, IgM for which the majority of cells will not have undergone affinity maturation and therefore will produce fewer false positive gene conversion events. Except for the PCR primers these sequences were obtained in the same manner as the chicken sequences in this paper, using the same enzymes for the same duration and rounds of replication on similar initial concentrations of RNA. Ten human IGHV genes were selected to calculate the maximum and minimum likelihood of false positive gene conversion events, including the most frequently rearranged genes and gene families with high genetic similarity. We used the IgM B cell repertoire sequences from healthy human controls 15 and the pipeline identical to the chicken repertoire gene conversion analysis described above. In the place of the pseudogene donor set, we used alleles in the same human V gene family (except for the IMGT/VQuest annotated V allele). For example, in analysing human IGHV3-23 sequences, IGHV3-23 was taken as the functional germline allele and other IGHV3 genes as the potential gene conversion donors. Results are shown in Supplementary Table 2.

### Statistical analysis

Visualization of gene conversion events was produced using BrepConvert, as described above. All other data were analysed using GraphPad Prism Version 9.1.0. Two-way ANOVA including multiple comparison Tukey tests was conducted to analysis the statistical significance in variation between the three tissue samples.

## Results

### V(D)J gene rearrangement selection preferences at the heavy chain loci

**Figure 2:**
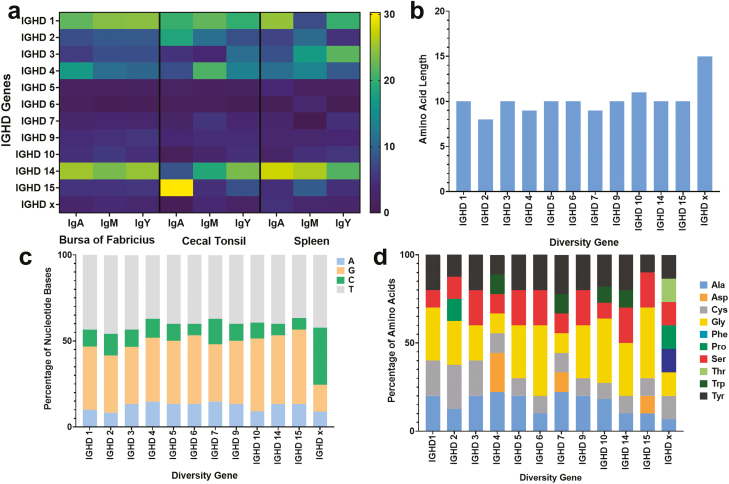
Analysis of IGHD gene usage and sequence analysis of each of the 12 unique IGHD genes. (A) IGHD gene usage across different antibody classes in multiple immune-associated tissues. The percentage usage of each IGHD gene, in each of the three antibody classes from each of the three tissues. Calculated as an average across all six birds studied. *N* = 99,904 sequences. (B) Comparison of amino acid length across all IGHD genes, (C) comparison of nucleotide composition across all IGHD genes, and (D) comparison of amino acid composition across all IGHD genes.

Diversity created during V(D)J gene rearrangement is limited by having just a single V gene and J gene, therefore analysis of IGHD gene recombination usage was conducted to identify if the full available genetic diversity is being utilized during this process. A total of 16 IGHD genes were identified by 26], only 12 of these gene segments (Supplementary Table 3) were analysed in this study as a number of them were found to be identical. These results show that there is an overall preference for IGHD genes 1 and 14, with IgA antibodies in the cecal tonsil showing a unique preference for IGHD 15 and a reduced preference for IGHD 14 (Fig. 2a). Analysis of the inferred genomic amino acid length of each IGHD gene from previous literature [26], and the nucleotide and amino acid composition were performed (Fig. 2b–d), with no particular feature giving evidence to explain the biased IGHD gene usage. Further investigation of the flanking RSS sequences (Supplementary Table 3 and Supplementary Fig. 2), also did not explain the biases seen for IGHD 1, 14, or 15.

### Effect of gene conversion on immunoglobulin gene diversity

**Figure 3:**
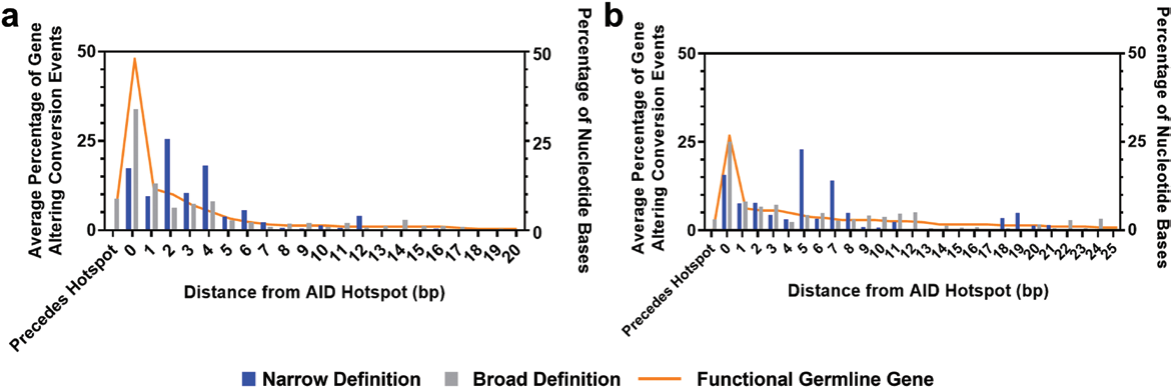
Comparison of the distance of minimum and maximum gene conversion events from an AID hotspot, against the distance to an AID hotspot from each nucleotide position in the single functional variable gene. In the (A) heavy chain loci using IGHV1-1*01 as functional gene, (B) light chain loci using IGLV1-1*01 as a functional gene. N: heavy chain gene conversion events (minimum and maximum) = 297,836, light chain gene conversion events (minimum and maximum) = 107,710. Distribution displayed as the average percentage of events at each distance averaged across six individual birds.

Gene conversion is the major factor in introducing genetic diversity into chicken immunoglobulins, therefore it was important to identify how the variable gene is altered. Identification of gene conversion events was limited to identifying events that caused visible genetic changes to the variable gene and were therefore denoted as gene-altering conversion events (GACEs); this distinction is important as SGC may incorporate silent changes that could not be detected. Due to high levels of similarity between the pseudogenes and the functional variable gene, the true definition of an insertion event, with the exact start and end points of each event are difficult to determine if silent changes occur at the 3’ or 5’ end of the GACEs. Therefore, each event was given a minimum and a maximum length. The minimum length was limited to the area that showed a visible genetic change from the functional gene, whereas the maximum length also included the area on either side of the minimum length that showed 100% genetic similarity between the functional gene and the donor pseudogene (Supplementary Fig. 3). Both definitions have been mapped across the variable gene in Supplementary Figs. 4 and 5. The number of nucleotide positions between the start of a gene conversion event and the end of an AID target hotspot was calculated for both the minimum and maximum lengths, the location of AID hotspots in both the heavy and light chain functional gene is displayed in Supplementary Fig. 6. This was compared to the distance between each nucleotide position of the functional variable gene and AID hotspots (Fig. 3). The minimum length was found to be closest to the true definition of an insertion event, as the maximum length of events follow the distribution seen in the functional gene and the maximum lengths of some gene conversion events were found to occur prior to the location of an AID hotspot. Therefore, the information recorded from the minimum length was used in all further investigations and analysis.

**Figure 4:**
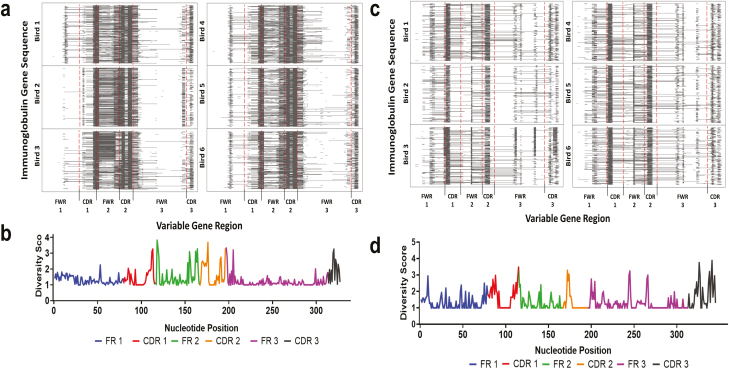
Visualization of the occurrence of gene conversion events across the length of the variable gene and diversity within the library of available pseudogenes. In the mapping of gene conversion, each row represents a different sequence, and each line represents the location and range of a GACE, the X axis denotes the six FWR and CDR regions of the immunoglobulin variable gene. Diversity score is calculated based on the genetic variation seen between the pseudogenes at each nucleotide position. (A) The location of gene conversion events in 1000 heavy chain repertoire sequences from each of the six birds studied. (B) The location of genetic variation in the chicken immunoglobulin heavy chain pseudogenes (*N* = 120). (C) Mapping the location of gene conversion events in 1000 light chain repertoire sequences from each of the study birds. (D) The location of genetic variation in the chicken immunoglobulin light chain pseudogenes (*N* = 49).

The location of minimum length GACEs that were identified in the variable gene region was visualized. Initial analysis found no differences in the location of these events between tissue samples (Supplementary Figs. 7 and 8), therefore sequencing data from all tissue types were aggregated for analysis and sequences from all tissues were represented in subsequent analysis. Analysis of the immunoglobulin heavy chain identified repeated patterns of insertion events across each individual bird studied, with all three CDRs, FWR 2, the 5’ of the FWR 3 and a small island in FWR 1 identified as ‘hotspots’ (Fig. 4a). The diversity created by gene conversion is a result of genetic variation available in the pseudogenes. To investigate the germline potential for GACE-induced diversity, a diversity score was calculated for each nucleotide position in the variable gene, based on the variation between the 120 known heavy-chain pseudogenes (Fig. 4b). This diversity analysis shows that the hotspots of variation in the pseudogenes matches the hotspots of GACEs. The pattern of GACE occurrence in the light chain loci is different from that of the heavy chain, with six smaller and more sporadically dispersed gene diversification sites (Fig. 4c). Despite occurring in different location hotspots to those seen in the heavy chain, these sites still appear to focus mainly on the CDR region, as might be expected. Similarly, a diversity score was calculated for each nucleotide position in the light chain variable region based on the 49 light chain pseudogenes (Fig. 4d). Like the heavy chain, the areas of genetic variability in the pseudogenes are homologous with the locations of genetic variability in gene-converted sequences. While the length and intensity of GACEs vary both within and between individuals across both the heavy and light chain immunoglobulin genes, the targeted ‘hotspots’ were found to be conserved in all individuals.

**Figure 5:**
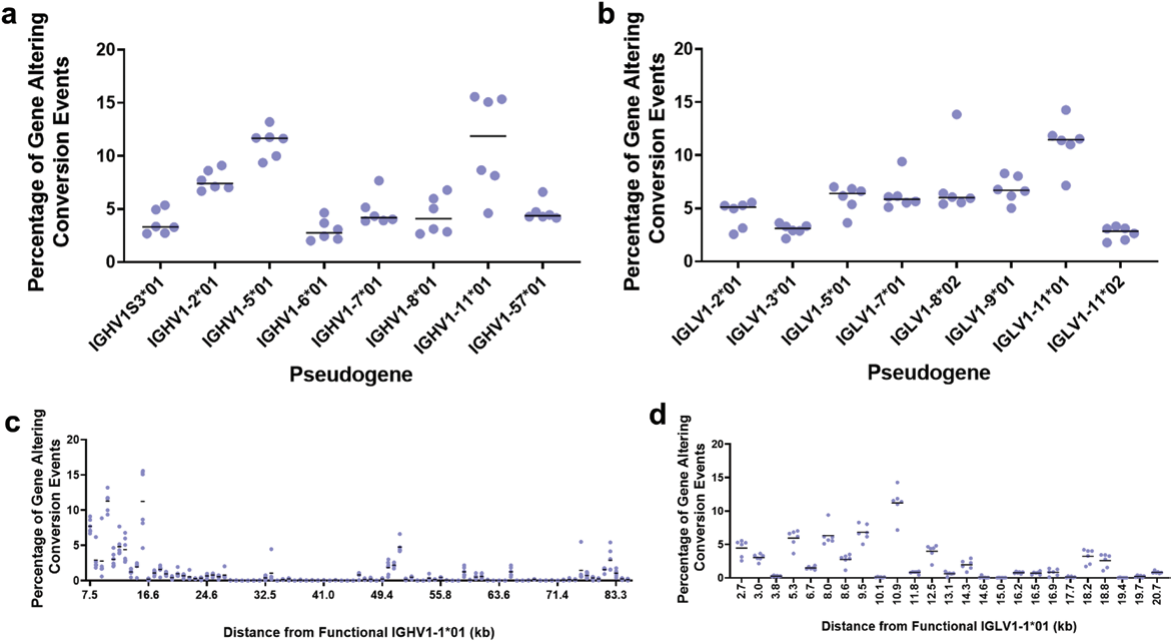
Comparison of the percentage usage of pseudogenes during GACEs. Each data point represents an individual bird, which was calculated using sequencing data aggregated from all tissue types. (A) Analysis of pseudogene selection in heavy chain repertoire sequences, displaying only pseudogenes that were present in more than 2% of gene diversification events in all birds. Heavy chain GACEs – *N* = 297,836. (B) Analysis of pseudogene selection in light chain repertoire sequences, displaying only pseudogenes that were present in more than 2% of gene diversification events in all birds. Light chain GACEs – *N* = 107,710. (C) Heavy chain pseudogene usage plotted against the pseudogenes distance (kb) from the functional variable gene (IGHV1-1*01). (D) Light chain pseudogene usage plotted against the pseudogenes distance (kb) from the functional variable gene (IGLV1-1*01).

Since gene selection preferences were identified during V(D)J gene rearrangement, we investigated whether there were any pseudogene selection preferences during gene conversion. Analysis of pseudogene selection in GACEs at the heavy chain loci suggests that a gene preference does occur. Pseudogene usage was calculated as a percentage of events in each bird, the percentages were then averaged across all birds, pseudogenes were labelled as ‘preferred’ if they occurred in more than 2% of GACEs. At the heavy chain loci, only 8 pseudogenes were identified as being preferentially selected for in all birds (Fig. 5a), and an additional 14 pseudogenes were identified as being preferred in one or more of the birds (Supplementary Table 4a). At the light chain loci, 8 of the 49 pseudogenes were shown to be preferred (Fig. 5b), with a further 15 pseudogenes identified as preferred in one or more birds (Supplementary Table 4b), while we did not see a relationship between pseudogene use, functional germline similarity or amino acid content. There was a correlation between pseudogene use and its germline proximity to the functional gene. 7 of the 8 heavy chain pseudogenes listed as preferred (Fig. 5a) are located at the 3’ end of the pseudogene region closest to the functional gene. The distance between each pseudogene and the functional gene in the germline is listed on the IMGT online database and measured in kilobases, this was compared to the percentage pseudogene usage. This analysis found that the heavy chain pseudogenes that were located proximally to the functional gene were selected at a higher rate than those located distal to the functional gene (Fig. 5c). No such correlation was identified in the light chain (Fig. 5d), however all of the light chain pseudogenes were found to be located within 20 kb of the functional light chain variable gene, which is the distance in which all preferred heavy chain pseudogenes are located. The potential influence of tissue type on pseudogene preference was investigated, however, the tissue of origin was found to have no effect on the pseudogenes that were selected during gene conversion (Supplementary Fig. 9).

**Figure 6:**
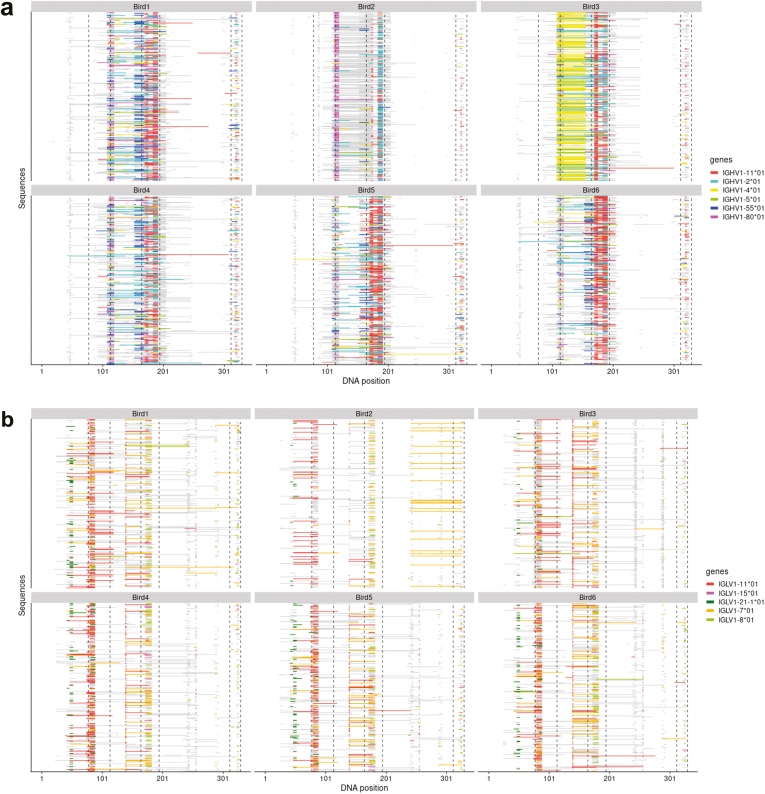
Visualization of pseudogene usage in gene conversion events across the variable gene region. Each individual bird analysis included sequencing data from all tissue types. (A) Mapping of the immunoglobulin heavy chain GACEs, colour coded by pseudogene usage in 1000 repertoire sequences. (B) Mapping of the immunoglobulin light chain GACEs, colour coded by pseudogene usage in 1000 repertoire sequences.

In-depth analysis of the pseudogene insertion sites revealed that certain pseudogenes are repeatedly inserted into the same location in the variable gene, with some patterns mirrored across all individual birds. Three pseudogenes that were preferred in all birds, two pseudogenes that were preferred in two or more birds, and one pseudogene that was preferred in only one bird as shown in Supplementary Table 4a and b, were selected for visualization (Fig. 6a and b). Comparison of pseudogene insertion patterns was not found to be affected by tissue of origin, so all sequences for each bird were collated resulting in the identification of three different patterns of pseudogene insertion. Firstly, it was noted that certain pseudogenes were being repeatedly inserted into a particular region of the variable gene in all birds. This was more apparent in the heavy chain as shown by pseudogenes IGHV1-11*01 and IGHV1-55*01 (Fig. 6a), this pattern was also seen in the light chain with pseudogenes IGLV1-5*01, IGLV1-11*01, and IGLV1-21-1*01 (Fig. 6b). The second pattern was that pseudogenes were being used in all birds, but the location of the insertion events varied between individuals. In the heavy chain, this is demonstrated by the use of IGHV1-2*01, being predominantly inserted into the FWR 2in birds 1, 4, 5, and 6, whereas bird 2 and 3 show greater use of this pseudogene in CDR 2 with limited insertions occurring in FWR 2. Similarly, insertion site changes can be seen for light chain pseudogene IGLV1-7*01. The third observation was that individual birds showed different preferences. This can be seen in the heavy chain where bird 3 shows a strong preference for the use of IGHV1-4*01 across FWR 2, a preference that is not seen in any other bird.

## Discussion

Knowledge of the ways in which chickens diversify their immunoglobulin genes is important for understanding how chickens respond to both vaccination and infection, which in turn will aid the development of effective vaccine programs. This study developed a novel method for the identification and assessment of SGC events in chicken immunoglobulin genes and identified repetitive patterns of diversification.

The findings of this study demonstrate that chickens, like many other species [27–30], display a gene selection preference in the early stages of B cell development during V(D)J gene rearrangement. The preferential selection of IGHD genes suggests that the selection of IGHD 1 and IGHD 14 (Fig. 2a) is presumably advantageous. However, the factors impacting this selection are still unknown and therefore further investigations need to be conducted to better understand this selection. Previous investigation has shown that the chicken variable region pseudogenes are directly followed by and fused to IGHD-like pseudogenes, and that during gene conversion the IGHD gene region is altered by these diversity region pseudogenes [31, 32]. Despite this potential caveat in the analysis of IGHD gene selection, the results of this study showed that there is a preference for production of IGHD regions that are genetically similar to that of IGHD 1 and 14, either via V(D)J gene rearrangement or gene conversion. IMGT currently only lists four chicken IGHD genes, however, in this study, a total of 12 chicken IGHD genes were analysed. These additional IGHD genes were included in this study because all 12 genes were identified within the immunoglobulin dataset generated in this study and have been listed in peer-reviewed sources [18, 26].

A caveat of this study was the potential for the occurrence of template jumping during PCR amplification, which together with misinterpretation of mutations as gene conversion events by BrepConvert could overestimate the numbers of gene conversion events seen. Since starting concentrations of RNA and numbers of rounds of replication can affect PCR template jumping errors, a UMI was assigned to each unique RNA prior to amplification. The collapsing of multiplicated UMIs into a single CCS reduces the possibility of identifying template jumping errors, but the occurrence of these errors can never be completely ruled out as a result of the genetic similarity between the pseudogene donor sequence and the functional gene in the regions flanking GACEs. To investigate the likelihood of misinterpretation of mutation for SGC events by the BrepConvert package, we used BrepConvert to analyse sequences of human immunoglobulin genes that were generated using the same laboratory-based methodology. Some gene families have more homology between family members and could be more prone to template jumping than others, so we looked at a range of common genes. We identified gene conversion-like events in 0.5–10% of our human IgM sequences, whereas in the chicken B cell repertoire, multiple gene conversion events were found to occur in 80% of sequences. Visualization of these events in human IgM sequences showed that these events occur in a much more randomized manner (Supplementary Fig. 10). Previous research by Duvvuri and Wu identified that gene conversion-like events occurs in 6% of human immunoglobulin sequences, which in the context of this study could reduce the false positive error rate of the BrepConvert package to around 5%. Analysis of gene conversion events in this study was limited to analysis of events that cause visible alterations to the genetic code. High levels of similarity between the functional gene and the available pseudogenes make it difficult to determine the exact start and end of each event as some of these insertions may be silent, which leads to each insertion event having two possible definitions, minimum length – limited to the length of sequence that was genetically different to the functional gene, and maximum length – which also includes the length of sequence either side of the minimum length event that showed 100% genetic similarity between the functional gene and the donor pseudogene. Previous work detailing the relationship between AID and gene conversion events [33, 34] enabled the determination that the minimum length was more appropriate (Fig. 3a and b).

Another caveat emerged from the analyses: 29–33% of the sequences inserted during gene conversion introduced an additional AID hotspot, which could be targeted by AID and become the site of additional insertion events, leading to subsequent misidentification of both donor pseudogene and events.

The key finding of this study was the identification of the predictable and repetitive manner in which gene conversion diversifies the chicken immunoglobulin heavy and light chain variable genes. These patterns were first identified when the location and range of each insertion event was mapped across the variable gene, highlighting three areas of the heavy chain variable gene and six areas of the light chain variable gene that are hotspots for genetic diversification (Fig. 4a and c). These repeated patterns were consistent across each of the six individuals studied, suggesting that these patterns are conserved across individuals. The location of gene conversion events in B cells collected from different tissues was compared, the result of which showed that the overall pattern of insertion is the same across all three tissues. Minor differences were found between tissues, however, these are more likely to be due to variation between individual sequences (Supplementary Fig. 7 and 8). Analysis of the occurrence of gene conversion in the CDR 3 region of the immunoglobulin heavy chain was limited due to the variation caused by IGHD and IGHJ gene recombination, which made it difficult to determine true gene conversion events. The patterns of gene conversion events identified in this study differ from those previously identified [32, 35], which state that gene conversion is limited to the CDR 3 region of the immunoglobulin variable gene. Differences in the conclusions of previous studies and those discussed in this study could be due to differences in the methodologies used in each study. Previous studies by [32] analysed less than 10 IGHV cDNA clones and [35] only analysed 5 IGLV sequences and were both focused on the CDR 3 region due to its role in the antigen binding site, whereas 43,000 IGLV and 99,000 IGHV sequences were analysed as part of this study, and the entire variable gene was studied. Additional studies investigating gene conversion have also discussed the occurrence of gene conversion events throughout the variable region gene [36–41].

Gene conversion was also found to be a largely restricted and conserved mechanism of diversification, due to the diversity available from the pseudogenes being underutilized in both the heavy and light chain loci (Fig. 4b and d), which were found to be preferentially selected during gene conversion. The discovery of restrictions during gene conversion is further supported by previous investigations by Refs. [35] and [42], proving the ability of the BrepConvert pipeline to correctly identify pseudogene donor sequences of gene conversion events. The preferential selection of a small number of pseudogenes at both loci (Fig. 5) combined with the repeated usage of the particular sections of genetic sequence from available pseudogenes (Fig. 6), contributes to the reduced level of diversification that occurs during this stage of B cell development.

Factors such as the age, breed, and the environment in which these chicks were raised may have impacted the level of diversity generated, as all six individuals studied were raised in the same specific pathogen-free laboratory environment and sampled at the same age. The sampling of Rhode Island Red chickens is a caveat of this study, as the sequences amplified from these birds were aligned in BrepConvert against germline variable region sequence that was downloaded from IMGT. However, this sequence was generated from a different breed of chicken, and the potential for differences in the genetic sequence of the two breeds could result in the misidentification of gene conversion events. In addition to this, there is also the potential for polymorphisms in the library of variable region pseudogenes, suggesting that pseudogene donors could be misidentified. Previous literature has stated that during B cell development antigens that are present in the gut are passed through the bursa of Fabricius, thus suggesting that exposing B cells to antigens early on in development aids the production of a diverse repertoire [43–45]. Therefore, further studies need to be conducted on more individuals from a wider variety of breeds and ages to identify if these patterns of gene conversion are truly conserved. Analysing birds that have been exposed to antigens either through vaccination or via infection would also provide crucial insights.

In summary, V(D)J gene rearrangement and somatic gene conversion, in 3-week-old Rhode Island Red chickens, produce an antibody repertoire of limited genetic diversity. We show that gene conversion is a highly conserved mechanism, which creates repetitive and predictable patterns of diversification with respect to gene usage and insertion sites, concluding that the diversity that is available during B cell development is not being utilized to its fullest potential.

## Supplementary Material

kyad002_suppl_Supplementary_Material

## Data Availability

The computational pipeline detailed above used to annotate gene conversion events is available as a R package ‘BrepConvert’. It is available at https://github.com/Fraternalilab/BrepConvert. Sequence data from this study is available at https://doi.org/10.5281/zenodo.6123269.

## Abbreviations

AID: activation-induced cytidine deaminase
CCS: consensus sequence
CDR: complementary determining region
CT: cecal tonsil
D: diversity gene
FWR: framework region
GACE: gene-altering conversion event
IMGT: immunogenetics information system
Ig: immunoglobulin
IgA: immunoglobulin A
IgH: immunoglobulin heavy chain
IGHD: immunoglobulin heavy chain diversity gene
IGHJ: immunoglobulin heavy chain joining gene
IGHV: immunoglobulin heavy chain variable gene
IgL: immunoglobulin light chain
IGLJ: immunoglobulin light chain joining gene
IGLV: immunoglobulin light chain variable gene
IgM: immunoglobulin M
IgY: immunoglobulin Y
J: joining gene
NGS: next generation sequencing
PCR: polymerase chain reaction
PID: patient identifier
SGC: somatic gene conversion
SHM: somatic hypermutation
UDG: uracil DNA glycosylase
UMI: unique molecular identifier
V: variable gene
