# Supplementary material for: Diversification of immunoglobulin genes by gene conversion in the domestic chicken (Gallus gallus domesticus)
Source: Discov Immunol. 2023 Jan 19;2(1):kyad002. doi: 10.1093/discim/kyad002 (PMC10917233; doi:10.1093/discim/kyad002)
Supplement: kyad002_suppl_Supplementary_Material [file kyad002_suppl_Supplementary_Material.pdf]

Supplementary Table 1: List of primers used during **A**) SMARTscribe[TM] reverse transcription **B**) PCR 1 and **C**) PCR 2.

a

Primer Name	*Primer Sequence*
SmartNNN	AAGCAGUGGTAUCAACGCAGAGUNNNNUNNNNUNNNNUCTT[rG][rG][rG][rG][rG]

b

Primer Name	*Primer Sequence*
Universal Smart20 Step Out	ggtagtcatgagtcgacactacactctatccgacaagcagt
IgA	ggtacctacagcctcaccac
IgM	gacttccagccctcctccatc
IgY	gaggaaaagcttcgtctgca
Light Chain (IgL)	tcaaaggaggagctgaacga

c

Primer Name	*Primer Sequence*	*Library 1*	*Library 2*
PID 1	ggtagtcatgagtcgacacta	Bird 1 Bursa	Bird 6 Bursa
PID 2	ggtagtatctatcgtatacgc	Bird 1 Cecal Tonsil	Bird 6 Cecal Tonsil
PID 4	ggtagacgtacgctcgtcata	Bird 1 Spleen	Bird 6 Spleen
PID 9	ggtagtcatgcacgtctcgct	Bird 5 Bursa	
PID 17	ggtagcacgtcactagagcga	Bird 5 Cecal Tonsil	
PID 22	ggtaggtgctgagcatcagac	Bird 5 Spleen	
PID 23	ggtagcactgatcgatatgca	Bird 2 Bursa	Bird 4 Bursa
PID 33	ggtagatacagcacagatgtg	Bird 2 Spleen	
PID 44	ggtagctcgatacgtgtagct	Bird 3 Bursa	Bird 4 Spleen
PID 45	ggtaggtgtctagacagctgt	Bird 3 Spleen	
IgA	aggacaacgaggagatgacc		
IgM	cttcatcagcaaatcggcca		
IgY	ttacagctgtagggtgaggc		
Light Chain (IgL)	gtcaagccacgagacctaca		

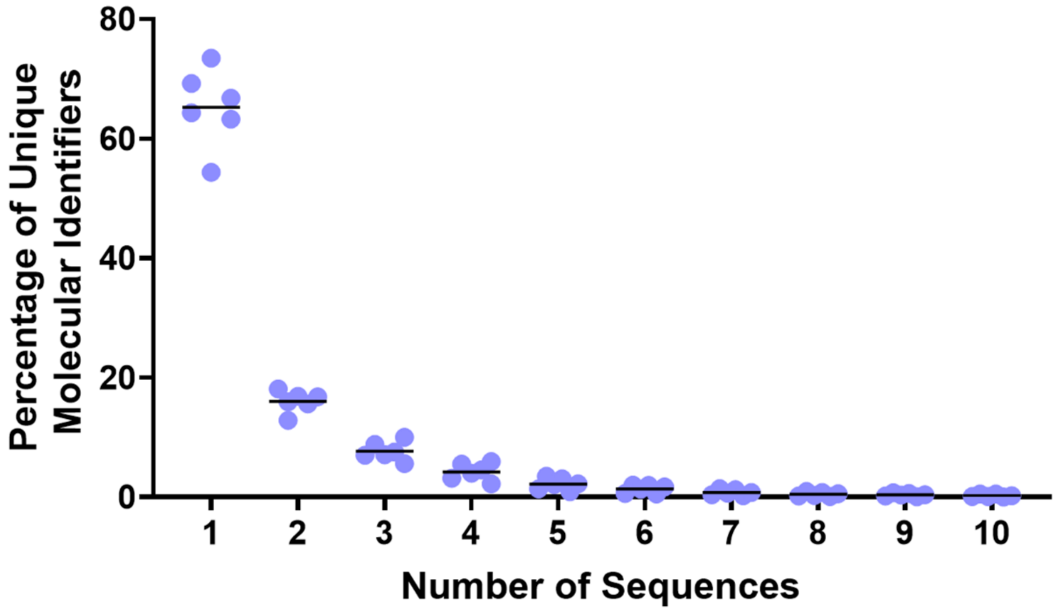

Supplementary Figure 1: Analysis of the number of sequences identified per unique molecular identifier (UMI). Each data point representing the percentage of UMIs in each individual bird.

Supplementary Table 2: Analysis of non-gene converting sequences using BrepConvert, calculating the rate of false positive identification of gene conversion events.

Germline Gene	*Number of Sequences Analysed*	*Percentage of False Positive Gene Conversion Events (%)*
IGHV1-2	3619	2.93
IGHV1-18	3365	1.63
IGHV1-69	6375	0.58
IGHV3-7	3754	10.00
IGHV3-23	7051	11.2
IGHV3-30	3919	5.28
IGHV3-33	3680	4.95
IGHV4-34	5784	1.66
IGHV4-39	6729	3.46
IGHV5-51	3134	0.77

Supplementary Table 3: Nucleotide sequences of the 12 unique IgHD genes discovered by Reynaud et al (1991) and analysed as part of this study.

Gene	*IGHD Nucleotide Sequence*	*RSS Nucleotide Sequences*
IGHD1	GGT TGT AGT GCT TAC GGT TGT GGT GCT TAT	1) GGATTTTGG 2) CACTGTG 3) CACGGTG 4) ACAAAAACC
IGHD2	GGT AGT GCT TGT TGT GGT CCT TAT	1) GGATTTTGG 2) CACCGTG 3) CACGGTG 4) ACAAAAACC
IGHD3	GGT AGT GCT TAC TGT TGT AGT GGT GCT TAT	1) GGATTTTGG 2) CACCGTG 3) CACGGTG 4) ACAAAAACC
IGHD4	GGT AGT GCT TAC TGT TGG GAT GCT GAT	1) GGATTTTGG 2) CACCGTG 3) CACAATG 4) ACAAAAACC
IGHD5	GGT AGT GCT TAC TGT GGT AGT GGT GCT TAT	1) GGATTTTGG 2) CACTGTG 3) CACGGTG 4) ACAAAAACC
IGHD6	GGT AGT GGT TAC TGT GGT AGT GGT GCT TAT	1) GGATTTTGG 2) CACCGTG 3) CACGGTG 4) ACAAAAACC
IGHD7	GGT AGC GCT TAC TGT TGG TAT GCT GAT	1) GGATTTTGG 2) CACCGTG 3) CACGGTG 4) ACAAAAACC
IGHD9	GGT AGT GGT TAC TGT GGT AGT GCT GCT TAT	1) GGATTTTGG 2) CACCGTG 3) CACGGTG 4) ACAAAAACC
IGHD10	GGT AGT GGT TAC TGT GGT TGG GGT GCT GCT TAT	1) GGATTTTGG 2) CACCGTG 3) CACGGTG 4) ACAAAAACC
IGHD14	GGT AGT GGT TAC TGT GGT TGG AGT GCT TAT	1) GGATTTTGG 2) CACCGTG 3) CACGGTG 4) ACAAAAACC
IGHD15	GGT AGT GGT TAC TGT GGT AGT GGT GCT GAT	1) GGATTTTGG 2) CACTGTG 3) CACGGTG 4) ACAAAAACC
IGHDX	GGT ACT TCT GGT GCC TGC ACC TTT TTC TAT CCT TCC TGC CCT TAT	1) GGATTTTGG 2) CACTGTG 3) CACGGTA 4) ACAAAAACC

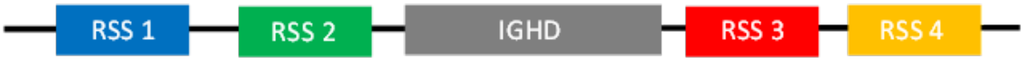

Supplementary Figure 2: Diagram displaying the location of the four RSS sequences in the relation to the IGHD gene.

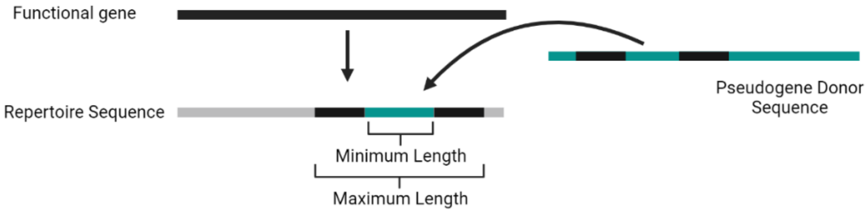

Supplementary Figure 3: Diagram displaying the minimum and maximum length of a gene conversion event. Minimum length being the length of sequence that is genetic altered from the functional gene sequences, maximum length being the length of sequence that has been altered as well as the length at the 5' and 3' end in which the donor pseudogene is identical to both the functional gene and the repertoire sequence.

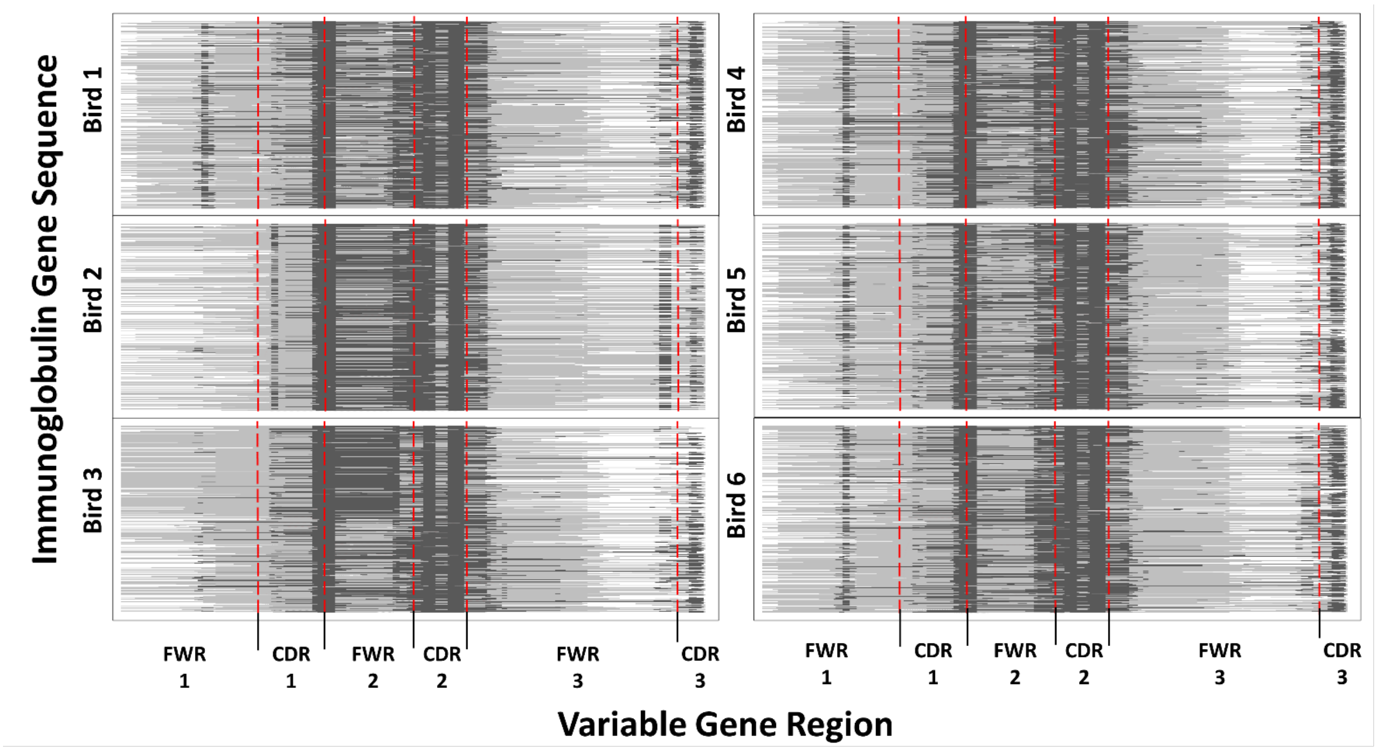

Supplementary Figure 4: Mapping the location of broad and narrow range GACEs from 1,000 heavy chain sequences from each of the study birds. Each row representing a different sequence, each black line represents the location and range of a narrow GACE, and each grey line represents a broad GACE. The X axis denotes the six FWR and CDR regions of the immunoglobulin variable gene.

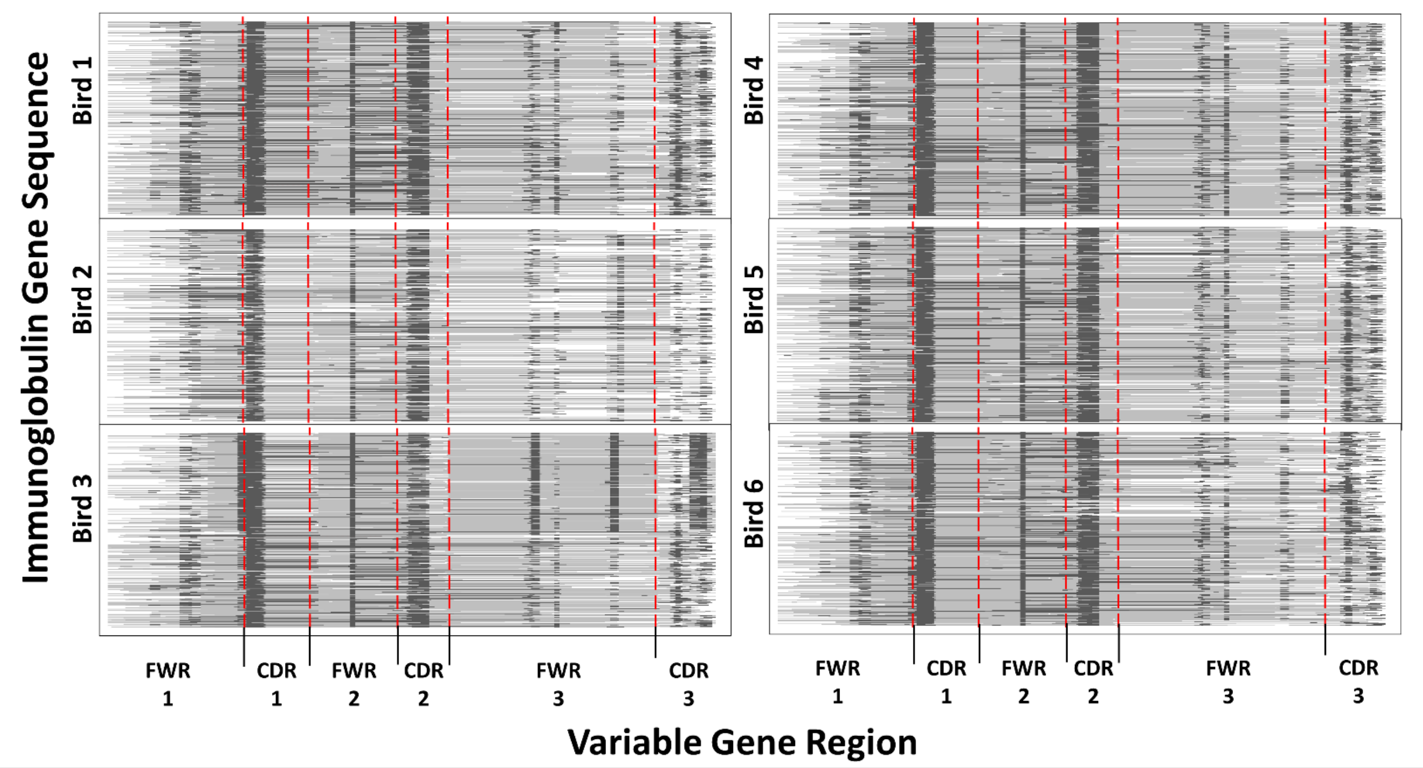

Supplementary Figure 5: Mapping the location of broad and narrow range GACEs from 1,000 light chain sequences from each of the study birds. Each row representing a different sequence, each line represents the location and range of a narrow GACE, and each grey line represents a broad GACE. The X axis denotes the six FWR and CDR regions of the immunoglobulin variable gene.

```
IGHV    gccgtgacgttggacgagtccgggggcggcctccagacgcccagaggagcgctcagcctc   60
        gtctgcaaggcctccgggttcaccttcagcagttatggcatgggttgggtgcgacaggcg   120
        cccggcaaggggctggagtgggtcgctggtattggcagcagtggtagtggcacagcatac   180
        gggtcggcggtgaagggccgtgccaccatctcgagggacaacgggcagagcacagtgagg   240
        ctgcagctgaacaacctcagggctgaggacaccggcacctactactgcgccaaagctgct   300
        ggt                                                            303

IGLV    caggcagcgctgactcagccgtcctcggtgtcagcgaacccgggagaaaccgtcaagatc   60
        acctgctccggggataggagctactatggctggtaccagcagaaggcacctggcagtgcc   120
        cctgtcactctgatctatgacaacaccaacagaccctcgaacatcccttcacgattctcc   180
        ggttccaaatccggctccacagccacattaaccatcactggggtccaagccgacgacgag   240
        gctgtctattactgtgggagtgcagacagcagcagtactgctg                    283
```

Supplementary Figure 6: The nucleotide sequence of the heavy and light chain functional genes (IGHV1-1*01 and IGLV1-1*1) as sated on IMGT online database [Lefranc et al., 2015]. The locations of AID hotspots marked by an asterisk (*) and the location of CDRs highlighted in grey.

Supplementary Table 4: The percentage usage of preferred heavy chain (a) and light chain (b) pseudogenes, with pseudogenes occurring in greater than 2% of GACE's highlighted in red. N: Heavy chain GACEs = 297,836, Light chain GACEs = 107,710.

a

	Bird 1	*Bird 2*	*Bird 3*	*Bird 4*	*Bird 5*	*Bird 6*
*IGHV 1S3*01*	2.3	1.7	1.1	1.7	1.1	0.8
*IGHV 1-2*01*	11.4	11.1	11.9	11.3	11.7	11.4
*IGHV 1-3*01*	5.4	7.2	5.9	5.6	6.5	6.2
*IGHV 1-4*01*	5.3	2.5	4.1	5.5	4.3	4.1
*IGHV 1-5*01*	8.9	8.8	9.8	8.9	9.1	9.5
*IGHV 1-6*01*	3.5	3.4	3.3	4.0	2.8	2.8
*IGHV 1-7*01*	3.4	6.1	3.1	3.8	3.0	3.1
*IGHV 1-8*01*	3.5	3.5	2.0	2.8	1.8	1.6
*IGHV 1-11*01*	4.5	3.4	4.7	4.9	8.1	8.5
*IGHV 1-35*01*	0.1	2.8	0.2	0.1	0.2	0.2
*IGHV 1-57*01*	3.1	5.4	3.6	3.0	2.9	3.2
*IGHV 1-69*01*	0.1	2.5	0.2	0.1	0.2	0.1
*IGHV 1-75*01*	0.4	4.1	0.4	0.6	0.5	0.4
*IGHV 1-80*01*	2.1	3.2	1.0	1.7	0.9	0.8

b

	Bird 1	*Bird 2*	*Bird 3*	*Bird 4*	*Bird 5*	*Bird 6*
*IGLV 1S8*01*	0.4	0.3	3.9	0.4	0.4	0.3
*IGLV 1S11*01*	0.5	0.4	5.2	0.3	0.5	0.2
*IGLV 1-2*01*	4.3	3.8	3.8	4.2	4.7	3.9
*IGLV 1-2*02*	7.7	14.9	5.0	7.7	7.6	7.8
*IGLV 1-5*01*	3.2	3.8	2.0	3.9	3.5	4.2
*IGLV 1-5*02*	2.9	1.4	1.9	3.3	3.0	3.8
*IGLV 1-7*01*	3.9	4.9	4.1	3.9	3.8	3.9
*IGLV 1-8*02*	7.9	3.7	12.2	7.2	7.1	7.4
*IGLV 1-9*01*	4.0	2.3	3.5	3.5	3.7	3.9
*IGLV 1-11*01*	6.2	4.3	8.0	6.5	6.3	6.6
*IGLV 1-11*02*	4.4	3.5	7.2	5.1	4.5	4.9
*IGLV 1-13*01*	4.4	3.3	3.3	4.0	4.7	4.3
*IGLV 1-15*02*	7.2	6.6	6.1	8.0	7.1	6.6
*IGLV 1-19*02*	2.8	2.8	2.3	2.9	2.9	2.9
*IGLV 1-20*02*	2.2	5.8	1.2	1.9	2.0	1.9
*IGLV 1-21*01*	2.6	1.9	1.9	2.8	2.9	2.8

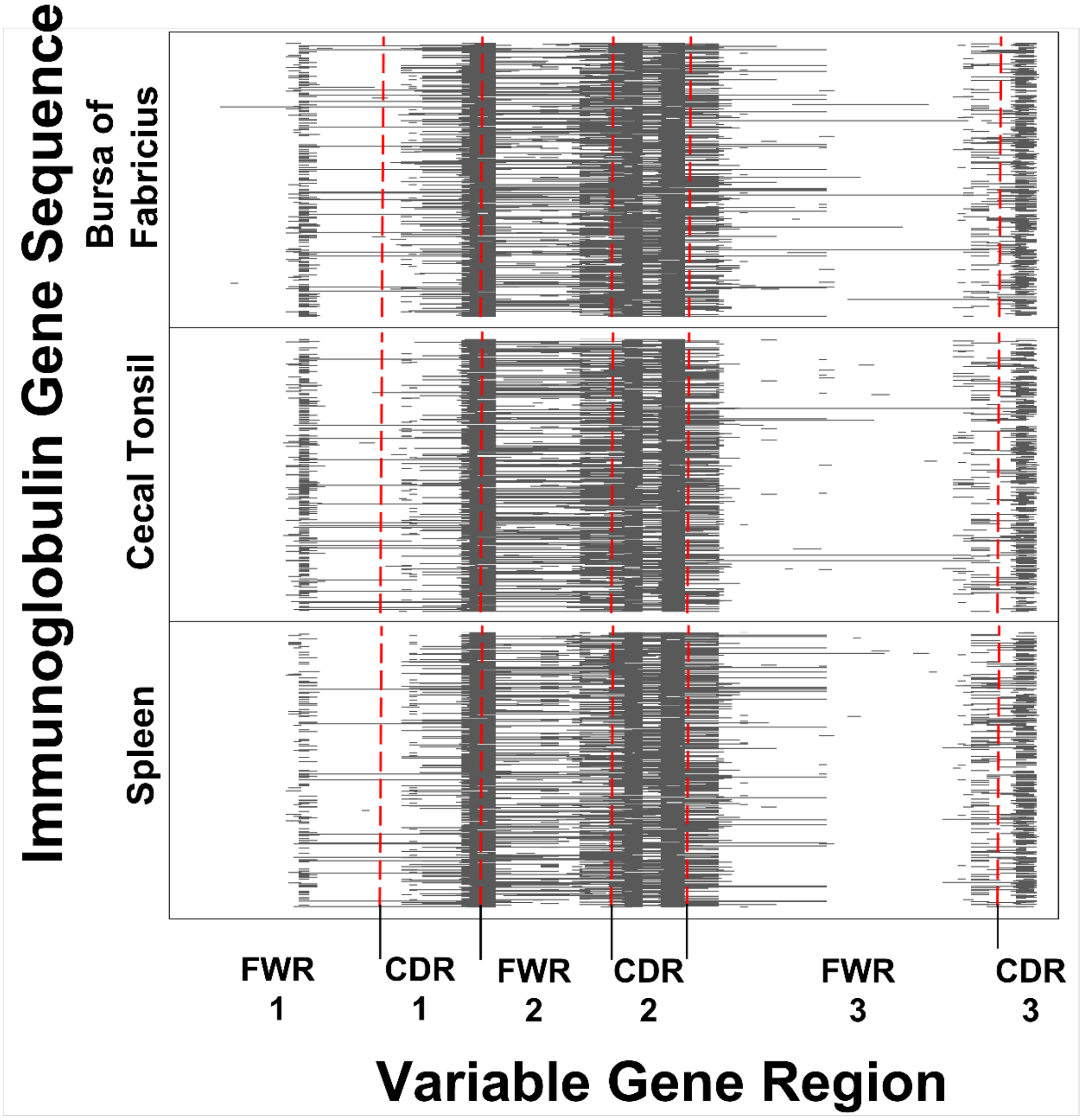

Supplementary Figure 7: Mapping the location of narrow range GACEs from 1,000 heavy chain sequences from each of the three tissues from bird 1. Each row representing a different sequence, each black line represents the location and range of a narrow GACE, and each grey line represents a broad GACE. The X axis denotes the six FWR and CDR regions of the immunoglobulin variable gene.

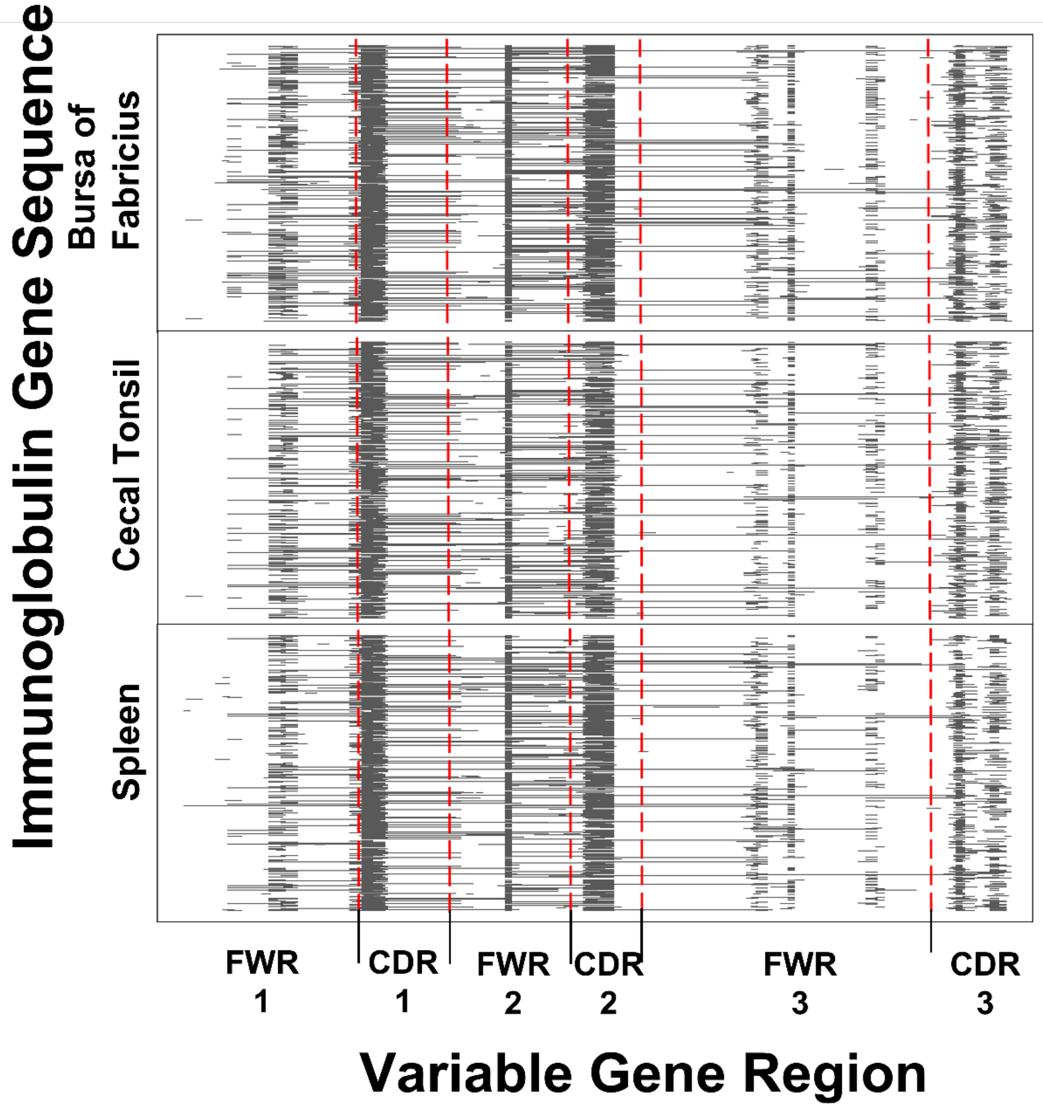

Supplementary Figure 8: Mapping the location of narrow range GACEs from 1,000 light chain sequences from each of the three tissues from bird 1. Each row representing a different sequence, each black line represents the location and range of a narrow GACE, and each grey line represents a broad GACE. The X axis denotes the six FWR and CDR regions of the immunoglobulin variable gene.

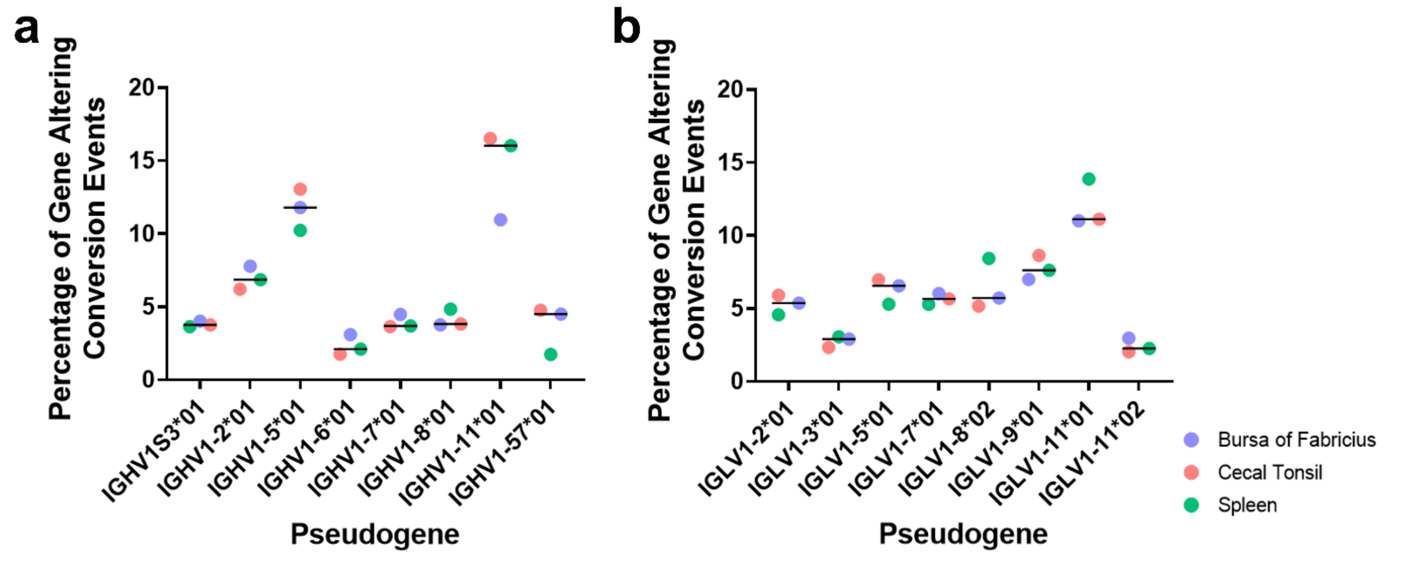

Supplementary Figure 9: Analysis of pseudogene usage was compared between sequences amplified from the bursa of Fabricius, cecal tonsil and spleen. Data point representing the mean percentage pseudogene usage within each tissue type, averaged across all 6 individual birds. **A**) Displaying the mean usage of heavy chain pseudogenes. **B**) Displaying the mean usage of light chain pseudogenes. Variation int the usage of each pseudogene between each tissue type was statistically analysed using a two-way ANOVA including multiple comparison Tukey test, but no statistical significance was found.

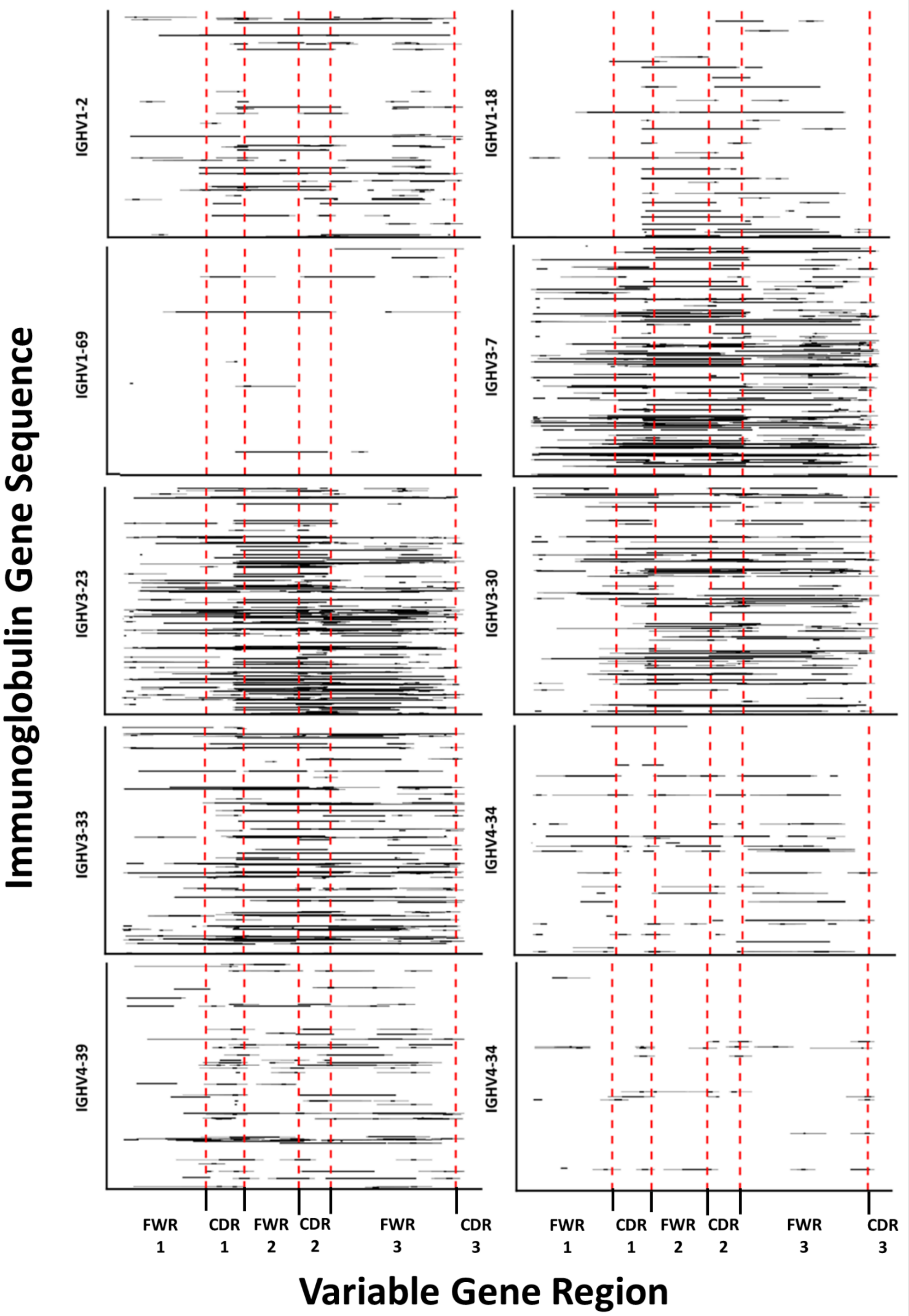

Supplementary Figure 10: Visualisation of gene conversion-like events in human IGHV repertoire sequences, as a result of template jumping, PCR crossover or misidentification of genetic mutations by the BrepConvert R package.
